# Gender Bias in Justice Evaluations of Earnings: Evidence From Three Survey Experiments

**DOI:** 10.3389/fsoc.2020.00022

**Published:** 2020-04-07

**Authors:** Carsten Sauer

**Affiliations:** Faculty of Political and Social Sciences, Zeppelin University, Friedrichshafen, Germany

**Keywords:** justice evaluations, just gender pay gap, gender inequalities, status beliefs, factorial survey, German-Germany

## Abstract

Previous studies in sociological justice research have found mixed results on the gender bias in justice evaluations of earnings. Some studies report a just gender pay gap favoring men; others do not find this gap. This study investigates the gender bias in justice evaluations by linking it to the inequality structure in which people are embedded. The empirical analyses are based on three factorial survey studies that consist of fictitious full-time employees with varying characteristics, including gender. One study was conducted with social sciences students, and two used population samples of German inhabitants. The results show that social sciences students revealed no gender bias in their evaluations. In the population surveys, both men and women showed a rating behavior favoring male employees. Respondents living in federal states with high actual gender pay gaps produced a larger bias favoring men. The findings indicate that actual inequalities between men and women influence the gender bias in justice evaluations.

## 1. Introduction

The *actual* gender pay gap captures the differences in earnings between men and women. The *subjective* gender bias in evaluations of earnings describes complementary differences in justice evaluations of men and women. While the existence of an actual gender pay gap is robustly documented for many countries, previous studies investigating the gender gap in justice evaluations of the earnings of men and women have yielded mixed findings. Jasso and Webster (1997) found a so-called just gender pay gap—the difference between earnings evaluated as just for male and female recipients—in a re-analysis of a factorial survey study conducted by Jasso and Rossi in 1974 (Jasso and Rossi, 1977). Male and female observers assigned higher just earnings to male recipients. In a later factorial survey conducted in 1995, using a student sample, they found only a marginal gap favoring women (Jasso and Webster, 1999). Jasso and Webster (1999) interpreted this finding in comparison to their previous study (Jasso and Webster, 1997) as a possible consequence of changing actual gender pay gaps over time.

I argue that the gender bias in justice evaluations of earnings is an *experience-based bias* that mirrors the gender inequality of the structural context in which individuals are mainly embedded. Distributive justice theories share the basic idea that similar individuals, based on socially defined and valued characteristics, expect similar rewards or earnings. Evaluators perceive justice if this condition is met, and they perceive injustice (either over-reward or under-reward) if this condition is not met because expectations are violated. The status value theory of distributive justice (Berger et al., 1972) and the justice evaluation theory (Jasso, 1978) highlight the importance of comparison processes within distributive justice judgements that rely on referential structures. These referential structures are general relations between a person's states of characteristics (in this case, male or female) and respective rewards (earnings) that are activated in justice evaluations. People who are embedded in a social structure that is highly gender unequal likely compare rewardees to generalized others (i.e., a typical female or male employee) that reproduce these inequalities. Thus, actors who experience gender inequality are more likely to activate a gender-biased referential structure in justice evaluations and therefore (unconsciously) perceive gender differences as legitimate. According to the mixed results of prior studies, university students who experience more gender equality will more likely activate a referential structure that does not produce gender bias, while members of the general population are more likely to experience gender inequalities over their life courses and reproduce them in their evaluations. If this is true, the difference between students and general populations reported in previous studies should still be detectable with more recent data, and the differences should be generalizable to other subpopulations that are prone to higher or lower gender inequality. In a first step, I therefore investigate whether gender biases still differ between students and the general population, including additional analysis by age and educational groups, and in a second step, I analyze whether differences can be detected between employees working in German federal states with more or less gender inequality.

The investigation of gender bias in earnings is important not only for justice research but also, more generally, for labor market sociology, as these biased attitudes have consequences for the actual behavior of labor market participants. For example, recently, it has become increasingly important to individually negotiate at least parts of one's earnings or other gratifications. In these negotiations it is on the one hand important for employees to formulate claims that yield an appropriate outcome, and on the other hand, supervisors have to evaluate these claims as legitimate. In the negotiation literature, it can be seen that a systematic gender bias is inherent (Dittrich et al., 2014; Kugler et al., 2018), partly because both negotiation parties likely exhibit a double standard for men and women. Discovering the mechanisms behind why people perceive certain income levels to be appropriate or fair for male and female employees sheds light on these processes. Experience-based gender bias questions the appropriateness of the accountability principle usually used to identify the fairness of individual negotiations.

The contribution of this paper is to apply the theoretical explanations offered by Berger et al. (1972) and Jasso (1978) to derive hypotheses about the direction and size of a just gender pay gap in observers' evaluations. By linking gender bias to structural inequality, it generalizes differences between students and the general population and provides tests for other sub-populations that likely produce more or less gender bias in their judgments—i.e., employees working in federal states with high or low gender inequality. Empirically, this is the first study that compares student samples and population samples using similar tools to detect gender biases that allow for the testing of differences for the first time. It therefore provides a continuation of the research initiated by Jasso and Webster (1997, 1999) with new empirical evidence.

To detect gender biases in justice attitudes, it is necessary to first use a method that allows to find gender gaps. The data collection method used here is a factorial survey design (Rossi and Anderson, 1982; Jasso, 2006), in which respondents evaluated so-called vignettes that described persons varying in multiple characteristics, including gender and gross earnings. These vignette-based justice evaluations can be used to measure the independent impacts of the recipient's gender and other characteristics on the justice evaluations of observers. With respect to this feature, factorial surveys have an advantage over justice measures of individuals' own earnings, as gender can be modeled as uncorrelated with other recipient's characteristics, e.g., occupational status and gross earnings, which are correlated in the real world. Second, it is necessary to compare observers who are embedded in different inequality structures. The empirical analyses, therefore, draw on a sample of social sciences students and two population samples. While the factorial survey module of the student sample and one population sample were identical, the second population sample used a different module and is used to emphasize the robustness of the findings. The social sciences students are embedded in a structural context in which relevant resources are not (or less) correlated with gender, and therefore, gender is unlikely to become a status characteristic in their daily interactions. The respondents in the population samples were sampled in different regions in Germany with differing degrees of earnings inequality between men and women. Thus, it is possible to investigate justice evaluations of people embedded in differing gender inequality structures. The following sections provide the theoretical background of the paper and then introduce the data and present and discuss the findings in light of the literature.

### 1.1. The Justice Evaluation Process

Questions surrounding distributive justice are part of the research program of the empirical sociological justice literature (Jasso et al., 2016; Liebig and Sauer, 2016) that has been developed over the last 50 years and now has a formalized core mapping the evaluation process. Distributive justice research distinguishes between *reflexive* and *non-reflexive* justice evaluations (Jasso, 2007). In reflexive justice evaluations, people evaluate their own rewards (*observer* = *recipient*); in non-reflexive justice evaluations, people evaluate the rewards of others (*observer*≠*recipient*). Previous studies on reflexive justice find a gap between the evaluations of men and women, with men expecting higher wages than women (Liebig et al., 2011, 2012; Valet, 2018). However, reflexive justice judgments are based on individuals' own outcomes and are therefore driven by two forces, justice deliberations and self-interest (Younts and Mueller, 2001). For example, only a small fraction of people evaluate themselves as being overpaid (Sauer and Valet, 2013). The impartiality (Jasso et al., 2019) of these reflexive judgments is therefore hardly given. Non-reflexive judgments, on the other hand, are not affected by conflicts of justice perceptions and individuals' own interests because people judge rewards by which they are not affected (especially when people evaluate fictitious others, as is the case in factorial survey studies). Non-reflexive judgments are, therefore, well suited to investigate justice attitudes and unconscious gender bias in judgments [for a review of the research on non-reflexive justice attitudes using factorial surveys, see Liebig et al. (2015)].

Following justice evaluation theory, in justice evaluation processes, people compare actual rewards to rewards perceived as just or fair (Jasso, 1978, 1980, 1986)^1^. The formalized evaluation can be stated as follows (Jasso, 1978):

(1) $$\begin{array}{r} J=\text{ln}\left(\frac{A}{C}\right)=\text{ln}A-\text{ln}C. \end{array}$$

The justice evaluation *J* of an observer is equal to the logarithmic ratio of the actual rewards *A* and the just rewards *C* of a recipient. The specification assumes comparisons as a central mechanism within justice evaluations. The actual rewards (gross earnings) are directly given, while the just gross earnings are a hypothetical value observers regard as just for given recipients. The just earnings depend on the levels of characteristics observers perceive as important. However, the specification leaves exogenous the substantive content of the just reward function (Jasso, 1980). Jasso and Wegener (1997) specify that the just reward depends on reward-relevant factors *x*, their weights and their combination. Thus,

(2) $$\begin{array}{r} C=h\left(x_{1},x_{2},\cdots \;,x_{n}\right). \end{array}$$

To learn about the content of these factors, theories that provide substantive predictions are useful. This study focuses on the relevance and weight of gender in justice evaluations; thus, predictions about reward-relevant characteristics are required^2^. Reward-relevant characteristics are those that entitle someone to receive a certain amount of rewards. These characteristics can be achieved, such as performance, or ascribed, such as gender (Berger et al., 1977). If these characteristics have a status value, they can be defined as status characteristics (Berger et al., 1977). Status characteristics divide trait carriers into status-high and status-low individuals and entitle status-high individuals to receive higher rewards. The status value is not an intrinsic feature of a characteristic (in this case, gender) but attached to the characteristic by generally shared beliefs. Reward expectations theory connects status characteristics to reward expectations and perceptions of justice and injustice (Berger et al., 1985). Reward expectations are formed based on status characteristics and a referential structure. Berger et al. (1985) distinguish three types of referential structures: categorical referential structures are based on “who you are,” ability referential structures are based on “what you can do,” and performance-outcome referential structures are based on “what you have done.” Reward expectations theory implies that categorical, ability and performance-outcome characteristics may together determine reward expectations and therefore justice evaluations.

Thus, status characteristics that refer to categorical differences, abilities or inputs are relevant for the observer to estimate the just earnings *C* of a recipient. Assuming this evaluation process, the justice evaluation stated in Equations (1) and (2) contains three types of characteristics: categorical variables, abilities and inputs. Gender is a categorical difference between recipients. If gender has status value in the eyes of the observer, it will be relevant in the justice evaluation process. It is assumed that the gender gap in just wages found in earlier studies (Jasso and Webster, 1997; Jann, 2008) occurred because gender had a status value, dividing people into status-low and status-high groups. On the other hand, if gender has no status value in the eyes of the observer, it is not a relevant factor for the justice evaluation. The observers produce in this case no just gender pay gap. In other words, the existence, sign and size of a just gender wage gap is connected to the status value of this characteristic. This can be written in a formal equation as follows:

(3) $$\begin{array}{r} J=\beta_{1}gender+\ldots +\beta_{n}\text{ln}A. \end{array}$$

The term *C* in Equation (1) is now replaced by characteristics that might be relevant for the justice evaluation, including gender. *J* is a function of the actual earnings (*A*) and the characteristics being evaluated as relevant for the assessment of the just reward. The question is now how inequalities between men and women influence the existence (β_1_≠0), sign (β_1_≶0) and size of a just gender pay gap. To link the justice evaluation process to the structural context, a closer examination of the referential structure of comparisons in justice judgments is in order.

### 1.2. Referential Structures in Comparison Processes

Early formulations of justice evaluation processes identified comparisons as the key mechanism how actors assess the justice or injustice of their rewards. The equity principle states that relative equivalence of two actors' ratios of inputs and outputs ensures perceptions of equity or justice in the eyes of the beholders. However, following the work of Berger et al. (1972), judgements based on comparisons between two individuals are not justice evaluations (e.g., both individuals could be underpaid). It is crucial to obtain a stable referential structure in which the comparisons are embedded. This means that people compare the rewards of specific people (either themselves in reflexive judgments or others in non-reflexive judgments) to a generalized other that represents a typical other for the specific comparison, e.g., a car mechanic or a teacher at a public school. The evaluator assesses then whether the outcome is just or unjust and if it is too high or too low. The rewards of the generalized other represent the typical earnings of similar people, while the normative evaluation of whether earnings are too high or too low is located in the comparison between the actual outcomes and the referential outcomes. Because in Germany, as in many other countries, the gender differences are remarkably high (more on this below), it is likely that gender is perceived as a status characteristic that is attached to higher earnings for men. Thus, the referential structure of individuals in an unequal population is likely to have a gender bias favoring men. Given the assumption that the process can be defined as a gender bias in the referential structure, it is likely that one will find gender gaps in just earnings in evaluators judgments who are themselves embedded in gender-unequal structures, while it is likely that people who experience less gender inequality do not have these biased structures.

Under the assumption of biased referential structures, it can be predicted under which structural conditions gender is likely to be a status characteristic and thereby a relevant factor in the justice evaluation process formulated in Equation (3). Under the structural condition of resource equality, it is likely that gender has no status value; therefore, gender is unimportant for the evaluation process. Status hierarchies are in this case not correlated with gender. In a subpopulation with resource equality, the justice evaluation of the observer should not be affected by the gender of the recipient. The hypothesis refers to the question of the existence of a just gender pay gap.

Hypothesis 1. *In a subpopulation with resource inequality (equality) between men and women, it is likely that male and female observers will (not) attach a status value to the characteristic gender of the recipient. Observers (do not) produce a just gender pay gap with their ratings*.

Under the structural condition of gender inequality, it is likely that gender has status value. If men are more likely to be resource-rich and women are more likely to be resource-poor, observers attach higher status to male recipients and assign higher earnings to the high-status group even though the recipients do not differ in other characteristics. This high-status group preference is shared by both the advantaged and disadvantaged groups, and accordingly, both male and female observers assign higher earnings to male recipients. The hypothesis refers to the question of the sign of a just gender pay gap. Hypothesis 2. In a subpopulation in which men earn on average more than women, it is likely that male and female observers will produce a just gender pay gap within their evaluations favoring male recipients.

### 1.3. Gender Inequality in Germany

The unadjusted gender pay gap is defined as the difference of the average gross earnings of men and women divided by the average gross earnings of men. Usually, the official statistics reporting the unadjusted gender pay gap use the arithmetic mean or the median of hourly or monthly wages of men and women. While the reported gap differs slightly depending on the measure used, the overall pattern is very similar. In Germany, the gender differences in earnings have remained persistently high over the last decade (Hobler and Pfahl, 2019) in comparison to other European countries. In the years 2008 and 2009, when the surveys of this study were conducted, the unadjusted gender pay gap of monthly median earnings in Germany was approximately 21% (see **Table 5**). Within Germany, the gender pay gap varies remarkably at the regional level. The second column of **Table 5** shows the pay gaps by federal state. In federal states located in West Germany (Schleswig-Holstein to Saarland) the gap varied between 18 percent and 28 percent, while in East Germany, the gap varied between 1 and 18% (Berlin included). Thus, there exist remarkable differences between federal states with the strongest divide between federal states located in the eastern and western parts of Germany. The adjusted gender pay gap (under the control of human capital factors and occupation) was approximately 8% (Finke et al., 2017) and remained also relatively stable over the last decade. Thus, people in Germany experience remarkable gender inequality in pay over the life course when they participate in the labor market.

While gender inequality is manifested in the German labor market, the situation is somewhat different for university students, especially social sciences students. The income students obtain for their monthly expenses is on average equal for female and male students (Isserstedt et al., 2010). Moreover, the student sample used in this study revealed no gender differences in study success (*mean*_*m*_ = 1.26; *mean*_*f*_ = 1.15; *T* = 1.27; *p* = 0.20; *n*_*m*_ = 697;*n*_*f*_ = 998) measured via self-assessment on an eleven-point rating scale (−5 to +5). The resource endowment (income and performance) was uncorrelated with gender, and it is therefore likely that gender has no attached status value in the referential structure. While students are undoubtedly socialized in a gender-unequal society and gender inequalities also exist at universities, the gender bias based on daily experiences should at least be lower than in other subpopulations. This is underlined by studies that investigate students transition to labor markets and their underestimation of gender discrimination in the workplace (Sipe et al., 2009).

## 2. Materials and Methods

To test the hypotheses stated above, it is necessary to first obtain heterogeneous respondent groups who experience varying degrees of gender inequality. I use data from one student sample and two random samples of the German population (the two population samples are independent of one another and differ in design and are therefore useful to demonstrate the robustness of the findings). Second, one needs an instrument that allows for the analysis of gender bias. Research shows that it is difficult to directly measure gender stereotypes due to social desirability bias and unconscious gender biases that people are unable to express directly. The factorial survey (Auspurg and Hinz, 2014) is a method that permits the detection of gender biases [and more generally sensitive topics, Auspurg et al. (2015)], especially in the case of justice evaluations of earnings (Gatskova, 2013; Auspurg et al., 2017). The following sections briefly describe the respondent samples and provide an overview of the factorial surveys and additional variables used and the analysis technique employed. There are methods reports available that provide additional information on the data used (Sauer et al., 2009, 2011, 2014).

### 2.1. Respondents

The university student survey (hereafter the *student sample*) was conducted during the summer term in 2008. Students in social sciences from 27 universities throughout Germany were interviewed via computer-assisted web interviews and computer-assisted self interviews in labs and in the presence of research assistants. The questionnaires consisted of the factorial survey module and additional questions on attitudes (after the factorial survey module) and questions on the socio-demographic background of the parents and students' personal situation. The analysis sample consists of 1,734 respondents.

The first population survey (*population sample 1*) was carried out in 2009 and consisted of randomly sampled respondents 18 years of age and older who were interviewed via computer-assisted personal interviews or self-administered interviews (paper and pencil or web interviews). The survey was conducted by a research institution with professional interviewers. The questionnaire consisted of the factorial survey module and additional questions on attitudes (after the factorial survey module) and questions on the socio-demographic background. As factorial survey studies go beyond standard questionnaires, the requirement in the computer-assisted personal interviewing version was to use experienced interviewers. Additionally, on 2 days, training courses were provided by the researchers to show the interviewers how the respondents had to rate the vignette task and how the interviewers had to behave as the respondents rated the vignettes and how to react in the case of questions. The analysis sample consists of 1,411 respondents^3^.

The data from the second population survey (*population sample 2*) were gathered in 2008 as part of a pretest of the German Socio-Economic Panel (SOEP; Schupp, 2009) via computer-assisted personal interviews. The program of the annual SOEP questionnaire for the following wave is pretested in each summer of the preceding year. The objective of these pretests is to test new modules and modifications of questions. Since 2002, the sample size has been approximately 1,000 respondents and considered representative of the German resident population 16 years of age and older (Siegel et al., 2009). There are two main differences between the pretest and the SOEP main survey. First, all interviews in the SOEP-Pretest are programmed as computer-assisted personal interviews, in contrast to the paper and pencil questionnaires mostly used in the main survey. Second, whereas the main survey is a study of private households, the SOEP-Pretest is a sample of individuals. The pretest sample is not related to the main SOEP, meaning that these respondents are not part of the panel study. The analysis sample consists of 952 respondents.

### 2.2. Factorial Survey

**Figure 1:**
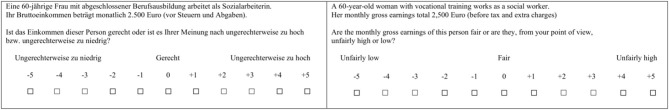
Example of a vignette with a rating scale used in population sample 1 and the student sample. The figure shows the German original version and the English translation by the author.

The factorial survey is a survey experiment that presents the respondents brief descriptions of persons or situations that consist of dimensions (e.g., gender, occupation, education) that vary experimentally in their levels. The vignettes of this study consisted of fictitious employees working full time (40 h per week). Each vignette provided information on at least the gender, age, education, and occupation of the recipient described, among other dimensions in more complex vignettes, together with gross earnings. In the terminology of Berger et al. (1972), the vignette dimensions are the characteristics of the recipient, and the gross earnings are the goal object. In the student sample and population sample 1, the number of dimensions (5, 8, and 12 dimensions) and the number of vignettes presented (10, 20, or 30 vignettes for each respondent) were varied in a between-subjects design^4^. Both studies used the same vignettes. An example of a vignette used is shown in Figure 1.

In population sample 2, a constant number of dimensions (10) and vignettes (24) was presented. This paper only focuses on five dimensions that were included in all studies^5^. Table 1 shows the dimensions and levels used for the analyses.

**Table 1:** Vignette dimensions and levels.

**Dimension**	**Levels**
Age	25, 35, 45, 55 years
Gender	Man, woman
Training	Without vocational degree, vocational degree, university degree
Occupation	Unskilled laborer, door(wo)man, locomotive engine driver, clerk, hairdresser, social work professional, computer programmer, electrical engineer, general manager, medical doctor
Earnings per month (Euros)	500, 950, 1,200, 1,500, 2,500, 3,800, 5,400, 6,800, 10,000, 15,000

The vignette samples were drawn via a quota design (D-efficient design) under exclusion of illogical or implausible cases (Dülmer, 2007)^6^. Illogical cases are, e.g., medical doctors without a university degree. The sampling technique ensured that the correlation of the gender characteristic and the other characteristics, e.g., occupation or gross earnings, was very low; therefore, no gender pay gap existed in the vignette samples. This is a mandatory requirement to investigate gender bias introduced by the respondents. Tables 2–4 provide information on the correlation structure of the vignette dimensions used for the analyses. The sampling procedure followed two steps: after sampling the vignettes, they were allocated to different decks (Jasso, 2006) that were randomly assigned to questionnaires that the respondents had to complete. The vignettes of the student sample and population sample 1 were additionally presented in random order for each respondent. This procedure ensures that potential method effects such as learning and fatigue (Sauer et al., 2011) are uncorrelated with substantive contents of the vignettes. Moreover, the respondents could skip vignettes if they did not want to answer. Population sample 2 was embedded in a large pretest, and it was not possible to randomize the order of the vignettes per person; thus, method effects regarding vignette order and substantive effects are not distinguishable. Moreover, it was not possible for the respondents to skip vignettes. The problem is described in greater detail in Sauer et al. (2009, 2014). Thus, interviews with less than 5 min of processing time for the vignette module (less than 12 s of processing time per vignette) were discarded from the analysis sample. The quality of the data from population sample 2 is therefore not as high as it is in the other two samples. Further details on the methodical setup of the factorial survey can be found in Sauer et al. (2009, 2011, 2014). Note that the data from population sample 1 were used for the analysis published in Auspurg et al. (2017) with a different approach and focus.

**Table 2:** Correlations of vignette dimensions for the student sample.

	**(1)**	**(2)**	**(3)**	**(4)**	**(5)**
(1) Gender	1.000				
(2) Age	−0.006	1.000			
(3) SIOPS	−0.022	0.040	1.000		
(4) Training	0.001	−0.002	0.202	1.000	
(5) Earnings per month (ln)	0.028	0.026	0.472	0.087	1.000

*SIOPS: Standard international occupational prestige scale*.

**Table 3:** Correlations of vignette dimensions for the population sample 1.

	**(1)**	**(2)**	**(3)**	**(4)**	**(5)**
(1) Gender	1.000				
(2) Age	−0.006	1.000			
(3) SIOPS	−0.035	0.035	1.000		
(4) Training	−0.006	−0.001	0.205	1.000	
(5) Earnings per month (ln)	0.022	0.021	0.476	0.086	1.000

*SIOPS: Standard international occupational prestige scale*.

**Table 4:** Correlations of vignette dimensions for the population sample 2.

	**(1)**	**(2)**	**(3)**	**(4)**	**(5)**
(1) Gender	1.000				
(2) Age	0.007	1.000			
(3) SIOPS	−0.006	0.036	1.000		
(4) Training	0.007	−0.036	0.250	1.000	
(5) Earnings per month (ln)	−0.009	0.018	0.538	0.144	1.000

*SIOPS: Standard international occupational prestige scale*.

#### 2.2.1. Rating Task

**Figure 2:**
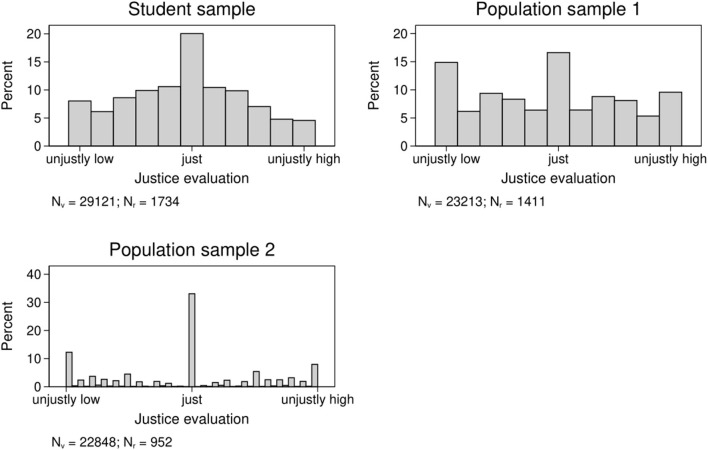
Distributions of justice evaluations by sample.

The respondents' justice judgments of gross earnings were obtained using two different rating procedures. In the student sample and population sample 1, respondents were asked to evaluate each vignette via an 11-point rating scale. The left extreme point (−5) was labeled “unjustly low,” the midpoint (0) was labeled “just” and the right extreme point (+5) was labeled “unjustly high.” The midpoint was coded as zero, the left segment as negative numbers, and the right segment as positive numbers. Population sample 2 used a three-stage rating task. First, respondents had to judge whether the earnings of a worker were just or unjust. If respondents rated the income as just, they were forwarded to the next vignette. If they rated the income as unjust, respondents judged in a second step whether the income was too high or too low. Third, the respondents stated the level of injustice on a 100-point scale. To achieve consistency with the two other samples—in which positive numbers indicate over-reward and negative numbers indicate under-reward—the ratings were transformed into a new scale in which perfect justice was coded as zero and the ratings that indicated under-reward were coded negatively. Thus, the new scale runs from −100 to 0 to +100. Figure 2 shows the distributions of justice evaluations by dataset.

In all surveys, the respondents had the opportunity to change their judgments of earlier vignettes when they compared them to later vignettes and had to adjust the ratings. This possibility was introduced in the description of the vignette task immediately before the first vignette. Moreover, in all survey modes, including computer-assisted personal interviews, the respondents self-administered their evaluations of the vignettes. In the computer-assisted personal interviews, the interviewers gave the laptop to the respondents and sat opposite them to preclude having the opportunity to view the evaluations.

#### 2.2.2. Context Variables

To test how actual inequality influences evaluations in the general population samples, the average earnings of full-time employees and the actual gender pay gap in different federal states in Germany were attached to the survey data^7^. There exist large regional differences in gender pay gaps across federal states. The lowest pay gap in 2009 for full-time employed people was measured in Saxony-Anhalt at 1 percent. The largest gap was measured in Baden-Württemberg at 28%. Table 5 provides the median earnings and gender pay gaps in 2009 for each state separately. Therefore, this context variable is useful to compare how the gender of the recipient influences justice evaluations of observers living in different federal states.

**Table 5:** Median monthly earnings and pay gaps by federal state in 2009.

**Federal state**	**Median earnings (Euro)**	**Gender pay gap (%)**
Schleswig-Holstein	2,502	18
Hamburg	3,079	20
Lower Saxony	2,598	24
Bremen	2,921	25
North Rhine-Westphalia	2,810	25
Hesse	2,959	23
Rhineland-Palatinate	2,688	22
Baden-Württemberg	2,941	28
Bavaria	2,779	25
Saarland	2,748	26
Berlin	2,510	18
Brandenburg	2,004	8
Mecklenburg-Western Pomerania	1,907	2
Saxony	1,931	10
Saxony-Anhalt	1,989	1
Thuringia	1,914	4
Total	2,648	21

*Source: Federal Statistical Office*.

### 2.3. Analysis

Each respondent rated several vignettes; therefore, the data have a multi-level structure. Because the assumption of uncorrelated error terms is violated and standard ordinary least squares (OLS) regression models would be biased (Cameron and Trivedi, 2009), the data were analyzed via multi-level regressions using a generalized least squares (GLS) estimator^8^. Note that alternative estimation with maximum-likelihood estimators leads to the same results.

The model in Equation (4) specifies that the justice evaluation *J* of vignette *v* of the *i*-th respondent is based on the given dimensions of each vignette. The outcome variable in the following regression models is the *z*-standardized justice evaluation per vignette. The independent variables are the five dimensions of gender (1 = male), age, education (dummy coded as follows: ref = without vocational degree; 1 = vocational degree; and 2 = university degree), occupation, and gross earnings. Occupation was transformed into a metric scale using the Standard International Occupational Prestige Scale (SIOPS; Ganzeboom and Treiman, 1996). Furthermore, according to the assumed evaluation process of Equation (3), the logarithmic representation of gross earnings was used. The regression Equation (4) displays the models with an attached intercept (β_0_), a respondent-specific residual (υ_*i*_) and an error term ϵ_*iv*_. Equation (4) was used to estimate the three models presented in Table 6.

(4) $$\begin{array}{r} J_{iv}=\beta_{0}+\beta_{1}gender+\cdots +\beta_{7}\text{ln}earnings+\upsilon_{i}+\epsilon_{iv}. \end{array}$$

**Table 6:** Multiple linear regression of justice evaluations of vignettes on vignette dimensions by sample.

	**Student sample**	**Population sample 1**	**Population sample 2**
Gender [1 = male]	−0.003	−0.068***	−0.074***
	(0.007)	(0.007)	(0.008)
Age	−0.018***	−0.024***	−0.019***
	(0.003)	(0.003)	(0.003)
SIOPS	−0.014***	−0.014***	−0.011***
	(0.000)	(0.000)	(0.000)
Without vocational degree	ref.	ref.	ref.
Vocational degree	−0.204***	−0.127***	−0.095***
	(0.008)	(0.008)	(0.010)
University degree	−0.300***	−0.198***	−0.132***
	(0.009)	(0.008)	(0.010)
Earnings per month (ln)	0.845***	0.888***	0.856***
	(0.004)	(0.004)	(0.004)
Constant	−5.816***	−6.154***	−6.129***
	(0.031)	(0.030)	(0.035)
*R*^2^	0.659	0.744	0.664
Vignettes	29,121	23,213	22,848
Respondents	1,734	1,411	952

*Standard errors in parentheses. SIOPS: Standard international occupational prestige scale*.

*p < 0.05, **p < 0.01, ***p < 0.001 (two-tailed t-tests).

Furthermore, I assume that the status value of gender differs between the population samples and the student sample. Additionally, both female and male respondents in the population samples are assumed to have similar status beliefs about gender. Equation (5) includes in addition to the gender of the vignette person (*gender*^*v*^) and the other dimensions, the gender of the respondent (*gender*^*r*^) and a cross-level interaction term. Equation (5) was used to estimate the results presented in Table 7.

(5) $$\begin{array}{r} J_{iv}=\beta_{0}+\beta_{1}gender^{v}+\beta_{2}gender^{r}+\beta_{3}gender^{v}gender^{r}\\ +\cdots +\upsilon_{i}+\epsilon_{iv}. \end{array}$$

**Table 7:** Multiple linear regression of justice evaluations of vignettes on vignette dimensions and gender of respondent by sample.

	**Student sample**		**Population sample 1**		**Population sample 2**	
	**(1)**	**(2)**	**(3)**	**(4)**	**(5)**	**(6)**
**VIGNETTE LEVEL**						
Gender^v^ [1 = male]	−0.003	0.009	−0.068***	−0.073***	−0.074***	−0.082***
	(0.007)	(0.009)	(0.007)	(0.009)	(0.008)	(0.011)
Age	−0.018***	−0.018***	−0.024***	−0.024***	−0.019***	−0.019***
	(0.003)	(0.003)	(0.003)	(0.003)	(0.003)	(0.003)
SIOPS	−0.014***	−0.014***	−0.014***	−0.014***	−0.011***	−0.011***
	(0.000)	(0.000)	(0.000)	(0.000)	(0.000)	(0.000)
Without vocational degree	ref.	ref.	ref.	ref.	ref.	ref.
Vocational degree	−0.204***	−0.203***	−0.127***	−0.127***	−0.095***	−0.095***
	(0.008)	(0.008)	(0.008)	(0.008)	(0.010)	(0.010)
University degree	−0.300***	−0.300***	−0.198***	−0.198***	−0.132***	−0.132***
	(0.009)	(0.009)	(0.008)	(0.008)	(0.010)	(0.010)
Earnings per month (ln)	0.845***	0.845***	0.888***	0.888***	0.856***	0.856***
	(0.004)	(0.004)	(0.004)	(0.004)	(0.004)	(0.004)
**RESPONDENT LEVEL**						
Gender^r^ [1 = male]	−0.052***	−0.036*	−0.005	−0.011	0.020	0.012
	(0.013)	(0.015)	(0.013)	(0.014)	(0.015)	(0.017)
**CROSS-LEVEL INTERACTION**						
Gender^r^ × gender^v^		−0.031*		0.011		0.015
		(0.014)		(0.013)		(0.015)
Constant	−5.795***	−5.801***	−6.152***	−6.150***	−6.138***	−6.135***
	(0.031)	(0.031)	(0.030)	(0.031)	(0.035)	(0.036)
R^2^	0.659	0.659	0.744	0.744	0.664	0.664
Vignettes	29,121	29,121	23,213	23,213	22,848	22,848
Respondents	1,734	1,734	1,411	1,411	952	952

*Standard errors in parentheses. SIOPS: Standard international occupational prestige scale*.

*p < 0.05, **p < 0.01, ***p < 0.001 (two-tailed t-tests).

**Figure 3:**
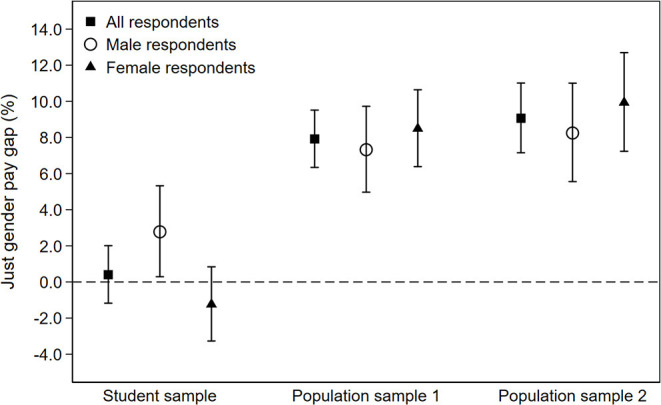
Just gender pay gap in percent (with 95% CIs) by sample and respondents' gender. Positive values indicate a gap favoring male vignette persons and negative values indicate a gap favoring female vignette persons. Evaluations differ between male and female students (*p* = 0.026) but do not differ in population sample 1 (*p* = 0.406) and population sample 2 (*p* = 0.360).

To illustrate the differences in evaluations between samples and male and female respondents the transformed b-coefficients estimated in Equation (5) will be presented in Figure 3. The figure shows how much more (in percentages) the fair earnings would be for male vignette persons compared to female vignette persons. The 95% confidence intervals (CIs) were calculated using the Delta method (Hole, 2007).

Additionally, the study assumes that there are differences between people living in federal states with high and low gender inequality. Thus, there should be an interaction effect between the vignette dimension gender and the actual gender pay gap in the federal state. Equation (6) includes the vignette dimensions, the structural context (the actual gender pay gap (*GPG*) and the average earnings per federal state), and the cross-level interaction between the vignette person's gender (*gender*^*v*^) and the gender pay gap in the federal state (*GPG*^*fed*.*state*^). The analysis sample was restricted to those respondents who were full-time employed because gender pay gaps were available only for full-time employees, so they directly experienced the difference in their daily interactions. The results are presented in Table 8. Additionally, the interaction effects were estimated separately for male and female respondents.

(6) $$\begin{array}{r} J_{iv}=\beta_{0}+\beta_{1}gender^{v}+\beta_{2}GPG^{fed.state}+\beta_{3}gender^{v}GPG^{fed.state}\\ +\cdots +\upsilon_{i}+\epsilon_{iv}. \end{array}$$

**Table 8:** Multiple linear regression of justice evaluations of vignettes on vignette dimensions and context variables by sample (full-time employees).

	**Population sample 1**			**Population sample 2**		
	**(1)**	**(2)**	**(3)**	**(4)**	**(5)**	**(6)**
**VIGNETTE LEVEL**						
Gender^v^ [1 = male]	−0.056***	0.031	0.031	−0.070***	0.014	0.014
	(0.011)	(0.038)	(0.039)	(0.014)	(0.041)	(0.041)
Age	−0.023***	−0.022***	−0.022***	−0.023***	−0.023***	−0.023***
	(0.005)	(0.005)	(0.005)	(0.006)	(0.006)	(0.006)
SIOPS	−0.015***	−0.015***	−0.015***	−0.012***	−0.012***	−0.012***
	(0.000)	(0.000)	(0.000)	(0.000)	(0.000)	(0.000)
Without vocational degree	ref.	ref.	ref.	ref.	ref.	ref.
Vocational degree	−0.121***	−0.122***	−0.122***	−0.093***	−0.093***	−0.093***
	(0.014)	(0.014)	(0.014)	(0.018)	(0.018)	(0.018)
University degree	−0.188***	−0.188***	−0.188***	−0.126***	−0.127***	−0.127***
	(0.014)	(0.014)	(0.014)	(0.018)	(0.018)	(0.018)
Earnings per month (ln)	0.910***	0.910***	0.910***	0.893***	0.893***	0.893***
	(0.006)	(0.006)	(0.006)	(0.008)	(0.008)	(0.008)
**RESPONDENT LEVEL**						
Gender^r^ [1 = male]	0.007	0.007	0.016	0.076**	0.076**	0.086**
	(0.021)	(0.021)	(0.024)	(0.026)	(0.026)	(0.029)
**STRUCTURAL CONTEXT**						
Average gross earnings of Fed. State	−0.230**	−0.230**	−0.230**	−0.254*	−0.253*	−0.253*
	(0.084)	(0.084)	(0.084)	(0.108)	(0.108)	(0.108)
Gender pay gap (GPG) Fed. State	0.010	0.030	0.029	0.029	0.049	0.048
	(0.039)	(0.040)	(0.040)	(0.049)	(0.050)	(0.050)
**CROSS−LEVEL INTERACTION**						
Gender^v^ × GPG Fed. State		−0.039*			−0.040*	
		(0.017)			(0.018)	
Female^r^: gender^v^ × GPG Fed. State			−0.034+			−0.034+
			(0.018)			(0.020)
Male^r^: gender^v^ × GPG Fed. State			−0.042*			−0.043*
			(0.017)			(0.019)
Constant	−5.736***	−5.777***	−5.783***	−5.782***	−5.825***	−5.830***
	(0.159)	(0.160)	(0.160)	(0.202)	(0.203)	(0.203)
*R*^2^	0.755	0.755	0.755	0.680	0.681	0.681
Vignettes	7,788	7,788	7,788	6,744	6,744	6,744
Respondents	483	483	483	281	281	281

*Standard errors in parentheses. SIOPS: Standard international occupational prestige scale*.

+p < 0.10 *p < 0.05, **p < 0.01, ***(two-tailed t-tests).

## 3. Results

### 3.1. Just Gender Pay Gap in Vignette Evaluations

The estimates of the regression models for the different respondent samples are presented in Table 6. First, the focus is on the effect of the gender of the vignette person on the justice evaluations for each sample. In the student sample, the effect of gender on the justice evaluation is insignificant, which indicates that minor importance is attached to this dimension. Students evaluated the justice of earnings of the vignette persons without a focus on whether the described person was male or female. The second model in Table 6 provides the estimates for population sample 1. The effect of the gender dimension is highly significant. The negative coefficient indicates that male recipients were more often evaluated as under-rewarded than female recipients. In other words, respondents produce with their ratings a just gender pay gap favoring men, as found by Jasso and Webster (1997). The third model in Table 6 provides the coefficients for population sample 2. As in the previous model, the effect of the gender dimension is negative, indicating rating behavior preferring male recipients. A test for different b coefficients of gender between the two population samples (*gender* × *sample*) with a pooled analysis reveals no statistically significant difference (χ^2^ = 0.83;*p* = 0.369), thus indicating a robust result due to its occurrence in two independent population samples. On the other hand, the tests between the student sample and population sample 1 (χ^2^ = 37.18;*p* < 0.001) as well as population sample 2 (χ^2^ = 49.61;*p* < 0.001) revealed significant differences.

The other coefficients and their interpretation are reported briefly as follows: the effect of a vignette person's age is negative and highly significant, meaning that older vignette persons were evaluated more often as under-rewarded than younger vignette subjects. This indicates that respondents reward seniority and potential work experience. The effect of the SIOPS has a significantly negative value, meaning that those vignette persons described by working in occupations with higher prestige scores were evaluated as more under-rewarded than those with lower scores (occupation status reward). The effects of vocational and university degrees are also significantly negative. The reference category is the dimension level without vocational degree. According to the respondents, the vignette persons who have a higher level of formal education should gain higher returns from their work (educational reward). Finally, the effect of gross earnings is positive: the more a vignette person earns, the more often respondents rated this person as over-rewarded, holding other dimensions equal.

In sum, age, education, occupation, and the associated earnings provided information on the recipients that all respondents used in their justice evaluation. There seems to be general agreement on the importance of these specific characteristics in justice evaluations of earnings; the coefficients are very similar. The only exception is the gender of the vignette persons, which was not important for students but crucial for the respondents in the two population samples. One must bear in mind that the vignettes in the student sample and population sample 1 were designed equally, so differences can be attributed to rating behavior and not to design elements. On the other hand, the rating task differed between population sample 1 and population sample 2; thus, their similar evaluation patterns indicate reliable results and a robust design.

### 3.2. Just Gender Pay Gap by Respondent Gender

The results in Table 7 provide information on the overall difference between respondents in the student sample and the population samples. To gain insights into whether these rating patterns were similar for both male and female respondents, as suggested by Hypothesis 2, respondents' gender was included in the regression. The models for the different samples are provided in Table 7. Models 1 and 2 report the coefficients for the student sample. Model 1 shows that the effect of respondents' gender on the justice evaluations is significantly negative, meaning that male students evaluated, on average, the vignettes as more unjustly low than female students. The interaction coefficient between the gender of the vignette person and the gender of the respondent in Model 2 indicates whether there were differences in rating behavior between men and women. The interaction effect is significantly negative, meaning that the rating behavior of male and female students differed with respect to the gender of the vignette person. Male students showed a tendency to favor male recipients (*b* = 0.021;χ^2^ = 4.10;*p* = 0.043), whereas female students showed an insignificant tendency to favor female recipients (*b* = 0.009;χ^2^ = 1.08;*p* = 0.299). Thus, male and female students did not account for gender similarly in their justice evaluations as it would be the case when it was a status characteristic for both groups. Models 3 and 4 show the coefficients for population sample 1. Model 3 indicates that male and female respondents evaluated the vignettes on average to an equal extent as being just or unjust. The interaction effect in Model 4 is insignificant, meaning that male and female respondents both produced to the same extent a just gender pay gap favoring male recipients in their evaluations. Models 5 and 6 show the coefficients for population sample 2. The results are very similar to those for the first population sample and are in line with Hypothesis 2.

Figure 3 shows the transformed b-coefficients of the regression models with the 95% confidence bars for each sample by gender. The graph highlights the different evaluation patterns between participants of the student sample and those of the two population samples. Moreover, it shows again high consistency of evaluations of the population samples.

### 3.3. Just Gender Pay Gap and Structural Context

To investigate how structural differences shape justice perceptions, the following analyses focus on the two population samples. The analysis was restricted to full-time employed respondents as they were directly affected by the actual gender pay gaps in the different federal states. The results are presented in Table 8. Models 1 to 3 show the coefficients for population sample 1. Model 1 includes the structural variables of average gross earnings and gender pay gap per federal state. The effect of average gross earnings is significantly negative, meaning that respondents living in federal states with high average earnings evaluated the gross earnings described in the vignettes more often as unjustly low compared to those respondents living in federal states with lower average earnings. This reflects differing referential structures with higher referential earnings of observers from high-income federal states. The gender pay gap in a federal state did not directly affect the justice evaluations. The second model includes the interaction term between the vignette person's gender and the gender pay gap in the federal state. The effect is significantly negative, meaning that the larger the gender pay gap in the federal state was, the larger the gender pay gap produced by respondents' ratings. The main effect of the vignette dimension of gender is insignificant, indicating that there was no gender bias in the evaluations if the actual gender pay gap was zero. The third model shows the coefficients of the three-way interaction with respondent's gender for male (χ^2^ = 6.17;*p* = 0.013) and female (χ^2^ = 3.60;*p* = 0.058) respondents separately. Again, the rating pattern was similar for male and female respondents (χ^2^ = 0.52;*p* = 0.472). Models 4 to 6 show the coefficients for population sample 2. The effects are very similar to those described above; again, the interaction effects in Model 5 and Model 6 are negative. Moreover, all coefficients are similar in both samples, even though the rating task was different, which indicates stable results.

### 3.4. Robustness of Results

One could argue that the actual gender pay gaps are especially salient for respondents who are actively participating in the labor market. Restricting the results presented in Tables 6, 7 to full-time employees yields similar results (as can also be seen in Models 1 and 4 of Table 8). Additional analyses with all respondents—not restricted to employed respondents—similar to those presented in Table 8 revealed mixed results. While the findings are reproducible with full population sample 2, they are not reproducible with full population sample 1 (gender pay gap of the federal state is statistically insignificant, although the coefficients have the same sign).

Moreover, student samples and general samples do not only differ by the structural conditions in which respondents are embedded. The main differences are that respondents in general samples are on average older and less well educated. Therefore, the findings presented above could reflect age or cohort as well as education effects. To test the robustness of the results of the models presented above, Table 9 shows the pooled analysis of the differences between the student and the population samples with restricted samples. The first model only considers respondents under the age of thirty; the second model restricts the analysis sample to respondents with a higher secondary school degree. In both models, there is a significant interaction effect between the gender of the vignette person and the subpopulation (student vs. non-student). The interaction effect eliminates the main effect of gender, meaning that gender is a relevant characteristic for young people or people with higher secondary education who are not students but has no impact on judgments when respondents are students. These findings resemble the results presented above and emphasize that it is likely that it is not the differences in age and education but the social contexts in which people are embedded and spend a crucial part of their lives.

**Table 9:** Multiple linear regression of justice evaluations of vignettes on vignette dimensions by age and education (all samples).

	**30 years and younger**	**Higher sec. degree**
**VIGNETTE LEVEL**		
Gender^v^ [1 = male]	−0.057***	−0.055***
	(0.013)	(0.010)
Age	−0.017***	−0.019***
	(0.003)	(0.002)
SIOPS	−0.013***	−0.014***
	(0.000)	(0.000)
Without vocational degree	ref.	ref.
Vocational degree	−0.195***	−0.182***
	(0.007)	(0.007)
University degree	−0.284***	−0.262***
	(0.008)	(0.007)
Earnings per month (ln)	0.831***	0.843***
	(0.003)	(0.003)
**RESPONDENT LEVEL**		
University student	−0.026	−0.017
	(0.016)	(0.013)
**CROSS-LEVEL INTERACTION**		
Gender^v^ × University student	0.056***	0.052***
	(0.014)	(0.012)
Constant	−5.723***	−5.795***
	(0.030)	(0.026)
*R*^2^	0.660	0.671
Vignettes	36,505	42,288
Respondents	2,103	2,434

*Standard errors in parentheses. SIOPS: standard international occupational prestige scale*.

*p < 0.05, **p < 0.01, ***p < 0.001 (two-tailed t-tests).

## 4. Discussion

This study investigated justice evaluations of earnings for male and female employees and linked them to actual inequalities. The goal was to explain the mixed results reported in previous studies on the just gender pay gap in non-reflexive justice evaluations (Jasso and Webster, 1997, 1999) by using predictions of sociological justice theories (Berger et al., 1972; Jasso, 1978, 1980; Jasso and Webster, 1997). The study assumed that actual gender inequalities lead to biased referential structures that typically associate men with higher earnings. The status value attached to male recipients reproduces gender inequalities in justice judgments of men and women. Thus, the direction and size of a just gender pay gap depends on actual inequalities people experience in their daily lives. The analysis was based on factorial survey studies conducted with one student sample and two population samples. The results show that male and female students did not produce a just gender pay gap with their evaluations. Social sciences students are an example of a more gender-equal subpopulation. In this population, it is less likely that gender has status value and therefore is not a relevant characteristic within the justice evaluation process. One must bear in mind that students are not only embedded in the structural context “university” but are also affected by socially shared attitudes toward gender in other contexts of social life. Therefore, they also experience gender inequalities in other contexts. However, their main arena of daily interactions in which status hierarchies emerge and spread is likely to be within the university with other students. As a limitation, gender equality may not apply to students in other subjects, as there could be differences that correlate with gender. The result is in line with previous research (Jasso and Webster, 1999) that also found only marginal differences in the ratings of male and female students. The difference is that in the previous study (Jasso and Webster, 1999), male and female students showed a tendency to favor female recipients.

The respondents of both population samples produced a just gender pay gap favoring male recipients. This gap was equal for male and female observers. The reason is that in a population with gender inequalities, it is likely that gender has status value and is therefore relevant in the justice evaluation process. Germany is a country in which a significant gender gap in earnings and income persists; therefore, the German population is an example of a structural context of substantial inequality between men and women. Although only a share of respondents participate in the labor market, these status differences are shared beliefs in wide parts of society because they have spread throughout the population. The fact that male and female respondents showed equal evaluation patterns is in line with findings in previous factorial survey research using a population sample (Jasso and Webster, 1997). Other factorial survey studies also found a gender gap in ratings (Jasso and Rossi, 1977; Alves and Rossi, 1978; Shepelak and Alwin, 1986; Jann, 2008; Adriaans et al., 2020).

The analysis of full-time employees resembled the findings of the complete population sample. Full-time employees directly experience inequalities in their goal-oriented daily interactions at their workplaces. There exist regional differences in the gender pay gap. The results show that the gender pay gap that observers experience influences their evaluations regarding the recipient's gender. Observers produced higher gaps in their ratings if they lived in federal states with a high actual gender pay gap. This evaluation behavior was measured for male and female full-time working observers in both population samples. The experienced structural inequalities between men and women affect justice attitudes toward gender. As these findings were replicated with two independent surveys, it is likely that these are reliable results.

A further note is that in all three datasets, there were similar effects for the other dimensions, indicating consensus regarding expected rewards for inputs and abilities such as education, occupation, and age. These findings are in line with the results of earlier studies (e.g., Jasso and Rossi, 1977; Alves and Rossi, 1978; Jasso and Meyersson Milgrom, 2008; Gatskova, 2013).

This research has limitations. First, it was assumed that people experience gender bias in their daily lives. However, future research could directly test the effects of daily interactions in the workplace or within an organization, as they are important for the emergence and spread of status beliefs and for justice evaluation processes. Therefore, not only data on justice perceptions but also information on the interactions of men and women in the workplace and organizations and on the inequality and power structures would be useful. Moreover, the comparisons between different subpopulations are based on cross-sectional data. The assumption is that contexts shape justice attitudes, meaning that students and employees change their attitudes as they come into other contexts. To test this underlying assumption, longitudinal data would be useful to separate changes in justice attitudes with respect to gender from differences between observers. It is likely that people change their attitudes when they leave the university and enter the labor market and unconsciously learn the new inequality structure and thereby change their referential structure. Therefore, research on this transformation process using survey experiments would be especially useful. Finally, the influence of gender inequality on justice evaluations was tested via regional pay gaps in Table 8. However, as the differences are mainly differences between East Germany and West Germany, one could also argue that the differences occur due to cultural differences between people who were socialized in different systems and societies (see, Lang and Groß, 2020). Future research could delve deeper into gender differences by taking into account family structures, motherhood (England et al., 2016), and household responsibilities. Research shows that gender inequalities in these dimensions at least partly contribute to gender differences in pay. It is likely that they also bias the justice judgements of observers, especially if the observers hold traditional norms regarding responsibilities in the household and family (e.g., male-breadwinner model; see, Lang and Groß, 2020).

Bearing the limitations of this study in mind, the findings provide important insights for sociological justice research, as they show how inequalities influence the justice evaluations of people. Moreover, the findings can be useful for inequality research, as justice attitudes reinforce actual inequalities. In all Western countries, levels of pay between men and women are only slowly becoming closer (Blau and Kahn, 2003, 2006). The legitimization of gender differences due to biased referential structures could be one reason for the slow reduction in the actual pay gap.

## Data Availability Statement

The population sample 2 is available for registered users at the Socio-economic panel (SOEP) at the German Institute of Economic Research (DIW). The data of the population sample 1, the student sample etc. are available from the author.

## Ethics Statement

Ethical review and approval was not required for the study on human participants in accordance with the local legislation and institutional requirements. Written informed consent for participation was not required for this study in accordance with the national legislation and the institutional requirements.

## Author Contributions

CS conducted the survey and wrote the paper.

### Conflict of Interest

The author declares that the research was conducted in the absence of any commercial or financial relationships that could be construed as a potential conflict of interest.

## Footnotes

^1^The theory is based on the research of Adams (1965), Homans (1974), Berger et al. (1972), and Jasso and Rossi (1977). Many articles provide discussions on different aspects of the theory [see Jasso (1978, 1980, 1981, 1986); Sołtan (1981); Markovsky (1985); Whitmeyer (2004)].

^2^The combination of these factors addresses interactions between gender and other characteristics, e.g., experience or education. The theory of double standards focuses on these interactions between job-related characteristics, such as competence and performance, and gender (Foschi, 2000); empirical research by Jasso and Webster (1999) shows that these double standards exist in justice evaluations.

^3^In the computer-assisted personal interviews, additional information was collected about the interviewer, e.g., work experience and training attendance, and the interview situations in interviewer questionnaires after each interview. With these pieces of information, it was possible to find those interviews that did not fulfill the requirements for the analysis: interviews were excluded from the analysis sample if respondents did not perform the task on their own but with the help of others. Moreover, three out of 81 interviewers had a tenure of one year or less and did not appear on one of the two training days. Their interviews were not used because they did not fulfill the requirements.

^4^The variations were part of a method experiment that investigated the effects of information load and fatigue during the interview. The results show only small effects of information load (number of dimensions) and fatigue (Sauer et al., 2011) that do not affect the results presented here. The respondents were randomly assigned to experimental splits.

^5^Due to the design, the correlations between the gender dimension and the omitted dimensions are very low, and the exclusion of other dimensions from the analysis does not affect the results.

^6^D-efficient designs (Kuhfeld, 2005) are built using a computer algorithm that specifies a sample characterized by a minimal intercorrelation between dimensions (main effects and interaction terms) while also ensuring a maximal variance and balance of the frequency of the dimensions levels. These designs ensure that the influence of vignette dimensions and interaction terms are mutually uncorrelated. In addition, the design features lead to minimal standard errors in data analyses and, therefore, in comparison to other designs (such as random samples), achieve higher statistical “power” and efficiency to reveal the influence of single dimensions. The D-efficiency for all vignette samples was above 90 (ranging from 0 to 100).

^7^The data on the average earnings of full-time employees are provided by the Federal Employment Agency (Frank and Grimm, 2010, p. 14). The gender pay gaps per federal state are provided by the Statistical Office of Rhineland-Palatinate (Schomaker, 2010a,b).

^8^The models were estimated with the statistical software Stata 13.1 (StataCorp, 2011). The user-written program estout (Jann, 2007) was used to format the tables.
